# A comparison of undergraduate outcomes for students from gateway courses and standard entry medicine courses

**DOI:** 10.1186/s12909-019-1918-y

**Published:** 2020-01-03

**Authors:** Sally Curtis, Daniel Smith

**Affiliations:** 1 Medical Education, Faculty of Medicine, University of Southampton, SO17 IBJ Southampton, England; 2 General Medical Council, London, NW1 3JN England

**Keywords:** Education, Medical, Undergraduate, Widening participation, Attainment, Outcomes, Progression and retention, UKMED, EPM decile, Situational Judgement test, Prescribing safety assessment

## Abstract

**Background:**

Gateway courses are increasingly popular widening participation routes into medicine. These six year courses provide a more accessible entry route into medical school and aim to support under-represented students’ progress and graduation as doctors. There is little evidence on the performance of gateway students and this study compares attainment and aptitude on entry, and outcomes at graduation of students on the UK’s three longest running gateway courses with students studying on a standard entry medical degree (SEMED) course at the same institutions.

**Methods:**

Data were obtained from the UK Medical Education Database for students starting between 2007 and 2012 at three UK institutions. These data included A-levels and Universities Clinical Aptitude Test scores on entry to medical school and the Educational Performance Measure (EPM) decile, Situational Judgement Test (SJT) and Prescribing Safety Assessment (PSA) scores as outcomes measures. Multiple regression models were used to test for difference in outcomes between the two types of course, controlling for attainment and aptitude on entry.

**Results:**

Four thounsand three hundred forty students were included in the analysis, 560 on gateway courses and 3785 on SEMED courses. Students on SEMED courses had higher attainment (Cohen’s *d* = 1.338) and aptitude (Cohen’s *d* = 1.078) on entry. On exit SEMED students had higher EPM scores (Cohen’s *d* = 0.616) and PSA scores (Cohen’s *d* = 0.653). When accounting for attainment and aptitude on entry course type is still a significant predictor of EPM and PSA, but the proportion of the variation in outcome explained by course type drops from 6.4 to 1.6% for EPM Decile and from 5.3% to less than 1% for the PSA score.

There is a smaller significant difference in SJT scores, with SEMED having higher scores (Cohen’s *d* = 0.114). However, when measures of performance on entry are accounted for, course type is no longer a significant predictor of SJT scores.

**Conclusions:**

This study shows the differences of the available measures between gateway students and SEMED students on entry to their medical degrees are greater than the differences on exit. This provides modest evidence that gateway courses allow students from under-represented groups to achieve greater academic potential.

## Background

Gateway courses, designed to attract students under-represented in medicine, are becoming an increasingly popular widening participation (WP) route into medicine in the UK [1]. Their aims are to provide entry to medical schools, not normally accessible without the highest grades in secondary education, for students with educational and social disadvantage and to support these students to succeed. There is a paucity of evidence on whether gateway courses achieve their aims and how gateway students perform compared to students on traditional, standard entry (SEMED) courses. This study is the first to compare the performance on entry and exit from medical school of gateway students with their SEMED counterparts.

Diversifying and enriching the medical profession by reducing social exclusivity is a key aim of the medical profession around the world [2–5]. Disadvantages faced by minority and under-represented groups applying to, and studying, medicine must be addressed to achieve this aim. Increasing the diversity of the profession and making it more representative of the population, especially with regard to socioeconomic status (SES), is high on the agenda of the UK Government, Medical Schools Council (MSC), National Health Service (NHS) and the British Medical Association (BMA) [6–9]. To attract under-represented students into medicine in the UK, much attention has been focused on raising aspirations and the development of more inclusive admissions processes to medical schools [1, 7]. The General Medical Council (GMC) have set a requirement in ‘Promoting excellence: Standards for medical education’ that processes of recruitment, selection and appointment of learners and educators are open, fair and transparent. To meet this standard medical schools must publish the criteria used for widening participation courses [10].

High academic attainment is a known barrier for admission to Higher Education (HE) and the medical profession. It is known that low SES negatively influences academic achievement, with WP students from such backgrounds being more likely to achieve lower grades [11]. In reference to under-representation of low SES students in HE, Gorard and See state: “If prior attainment … is used to determine future participation (and attainment), and because we know that SES and attainment are linked, then the situation we find is as expected” [12].

Many WP students have not had the opportunity to achieve their academic potential whilst at secondary school and sixth form/college and, therefore, do not have the opportunity to enter medical school. Traditionally, admission to medical school includes a strong weighting on high academic achievement, usually as A level qualifications (16–18 subject specific examinations in England, Wales and Northern Ireland; Scotland have Scottish Higher examinations), often alongside high scores in aptitude tests such as the UCAT (previously UKCAT). A levels are Graded A* to F, with A* being the highest grade and typically medical schools require three A levels with A* or A grades. Studies have reported academic attainment and UCAT scores to be significant predictors of medical school outcome [13, 14]. Mwandigha et al., who found UCAT scores added some predictive value in addition to secondary school achievement for undergraduate academic performance, supports this [15]. The ‘UKCAT-12’ study looked at the predictive validity of UCAT scores and educational attainment at 12 UK medical schools that had first year medical school exam results available for 2008 to 2010. UCAT scores provided small, but incremental, validity when taking prior educational attainment into account. The authors conclude their study confirmed the validity of using all the existing measures of educational attainment and aptitude in selection processes [16]. However, other single institution studies have reported no, or little, predictive value of the UCAT in the early years [17, 18]. Interestingly, Mwandigha et al. recently identified an inverse relationship between undergraduate achievement and the performance of the secondary school a student attended and suggested a reduction in academic entry criteria for those applying from lower performing secondary schools [15].

Six-year gateway courses aim to support students from educationally and socioeconomically disadvantaged backgrounds enter and progress through medical school. Gateway courses provide an additional year of study, either as an initial year before entering year 1 or integrated in the first two years of study. The additional year supports the transition into HE and provides a variety of skills and knowledge designed to help students succeed in their future studies. Many gateway courses provide clinical or community placements alongside modules in professionalism and science based topics, delivered in a highly supportive environment.

Contextual application processes assess the educational, social and individual context of students’ prior academic attainment alongside their potential to study medicine. Entry onto gateway courses usually employs contextual admissions processes whereby applicants fulfil course-specific eligibility criteria, sometimes including regional requirements, often alongside reduced A level grades. The typical A level grade requirements for gateway courses range from AAB - BBB and are frequently one to three grades lower than the requirements for the standard entry courses at the same institution.

It is important to recognise that the background context for some students on gateway courses, which prevented them from realising their academic potential before entering medical school, will continue to have a negative effect on their undergraduate studies [19]. Therefore, a comparison of their performance with students who do not share similar barriers does not provide a wholly representative picture of their achievements.

There is minimal evidence of educational outcomes and attrition rates of students on gateway courses reported in the literature [20, 21]. Mahesan et al. found that, following intercalation to undertake a BSc degree, gateway students at King’s College London attained Year 5 results, which were on average over 4 points lower than students on the 5-year standard entry to medicine course (SEMED) [22]. However, there has been no multi-site comparison of performance between students on SEMED and gateway courses.

Common undergraduate outcome measures used to determine performance on exit from medical school in the UK are the Educational Performance Measure (EPM) decile, the Situational Judgement Test (SJT) score, the Prescribing Safety Assessment (PSA) mark and the award of a medical degree *(these outcomes are further described in the methods section).*

Kumwenda et al. reported students from state schools achieve higher EPM scores than those attending independent schools, when entering medical school with similar grades [23]. This provides an interesting perspective on how well different school types prepare students for studying medicine but, as the authors of the study acknowledge, the findings need to be interpreted within the whole context of the individual’s educational circumstances: attending a state school does not always demonstrate educational and social disadvantage.

A small number of studies have reported graduate outcomes in relation to entry qualifications and aptitude test scores. McManus et al. reported that A-levels and aptitude tests such as UCAT demonstrate construct level predictive validity with undergraduate and postgraduate performance although this meta-analysis did not look at EPM, PSA or SJT as outcomes [24]. MacKenzie et al. have shown the UCAT total score is predictive of performance on the EPM and the SJT [25].

In 2017, Maxwell at al. reported on the PSA [26]. They found a statistically significant variation in performance between medical school cohorts and a strong positive correlation in performance for individual schools over two years. However, these studies looked at whole cohorts and did not compare SEMED with gateway students.

To address the gap in the literature this study compares the performance on entry of students on gateway courses with those on SEMED courses, and undergraduate outcomes after accounting for attainment and aptitude measured at entry.

## Methods

Data from three medical schools that run established gateway courses and SEMED courses are included in the analyses of this study. The institutions are King’s College London, The University of Southampton and Norwich Medical School at The University of East Anglia. These medical schools have the longest running gateway courses delivered alongside standard entry courses in the UK.

### Admissions processes 2007–2012

Entry to the gateway courses included in this study require the fulfilment of eligibility criteria in addition to academic and non-academic requirements. Fulfilment of the eligibility criteria, individual to each institution, aims to identify the educational and or social disadvantage of the applicants.

### King’s College London

During the time-frame of this study, entry requirements for the Extended Medical Degree Programme (EMDP) – a gateway MBBS course at King’s College London have undergone significant change.

A-level requirements were historically weighted according to the A-level performance of an individual applicant’s school, as compared to the national average. Between 2007 and 2011, applicants were typically required to achieve between AAB-BCC at A-level, including both Chemistry and Biology, and have obtained a minimum of grade C in GCSE English Language and Mathematics. In 2012, requirements increased to AAB-BBC, with a B in GCSE English Language and Mathematics.

By comparison, in 2007 applicants to King’s 5-year MBBS (SEMED) course were required to achieve AAB/C, including Chemistry and Biology; rising to AAB/B in 2008; AAA/B in 2009; and AAA in 2010. NB. Requirements for both King’s gateway and SEMED courses have again risen since the period included in this study (AAB and A*AA respectively).

UCAT scores were used from 2008, typically very minimally, as a final filter in cases where two applicants scored the same in all other elements of assessment. Until 2011, EMDP applicants were required to take the mental agility test (MAT) element of the Personal Qualities Assessment [27], organised by King’s at no cost to the applicant.

Academic requirements were used in conjunction with non-academic criteria such as an applicant’s commitment to community – as judged through personal statements and academic references – to determine invitation to interview. Panel interviews were replaced by multiple-mini interviews (MMIs) from 2010. Throughout, applicants were required to attend a non-selective state school within the respective target areas; have attended only non-selective state education since the age of 11; and have no prior degree experience.

The EMDP’s geographical target area also underwent significant change during the period under study: widening from three specific inner London Boroughs (Southwark, Lambeth and Lewisham, 2001–2007); to all 15 inner London Boroughs (2008–2010); to all 32 Greater London boroughs and Kent & Medway (2010–2012).

### The University of Southampton

Applicants to the BM6 gateway course at Southampton were required to achieve a minimum of BCC at A level to include Biology and Chemistry and five GCSEs A-C to include Mathematics, English Language, and Biology and Chemistry or additional science and science or combined science.

Applicants were required to fulfil two of the following five eligibility criteria: first generation Higher Education; parents or the applicant being in receipt of means tested benefit; being in receipt of the educational maintenance allowance (EMA); living in an area of the lowest 20% on the index of multiple deprivation (IMD) or a member of a travelling family; in care of the local authority.

Personal statements of eligible applicants were scored against non-academic criteria: (demonstrating they had learnt from their experiences of interacting with people in health or social care settings, were self-motivated, had initiative, were able to interact successfully with others, were literate and articulate) those with the highest scores were invited to interview. UCAT scores were not considered in the main selection process, but could be used as a differentiator if there were two or more applicants with the same score from selection days but only one place on offer. Interviews were scored against non-academic criteria and those achieving the highest scores were offered a place.

Applicants to the BM5 SEMED course were required to achieve a minimum of BBB at A level to include Biology and Chemistry and 7 GCSEs at grade B. Personal statements of eligible BM5 applicants were scored against non-academic criteria and those with the highest scores were offered a place at medical school.

Although all students are required to take the UCAT, as its use was under consideration, the scores did not contribute to the selection process between 2007 and 2012.

### Norwich medical school

The MBBS with a Foundation Year gateway course at Norwich Medical School took its first students in 2007. Applicants were required to achieve a minimum of BCC at A level although no subject was specified and five GCSEs at grade B to include Mathematics and English Language. Applicants were required to meet at least one of the following contextual criteria: Combined household income under £25,000 per year excluding Government benefits; have been in local authority care; meet POLAR 2 criteria*; have parents/guardians with no higher education qualifications; have parents/guardians who were either unemployed or their occupations fall within particular groups of the socioeconomic classification system (NS SEC 4–8). For 2009 entry, the minimum A level requirements increased to BBC and for 2012 entry, this increased further to BBB and six GCSEs at grade B to include Mathematics and English Language.

*The Participation of Local Areas classification (POLAR) is a UK-wide geographical measure of the proportion of young people, living in a particular area, who participate in HE by the age of 19. The areas are assigned to quintiles with the lowest (quintile 1) representing the areas of least participation of young people in HE and the highest (quintile 5) representing the areas of highest participation. POLAR 2 was used between 2007 and 2011 and has been updated twice since then, with POLAR 4 being the current measure.

The personal statement and reference sections of the UCAS application form of eligible candidates were scored against non-academic criteria (capacity for self-directed learning, capacity to work effectively in groups and with colleagues, capacity to take responsibility, personal effectiveness) for suitability to study medicine on a gateway course. Applicants with the highest combined screening score and UCAT score were invited for interview. The applicants with the best performance at interview received an offer of a place.

Applicants to the MBBS SEMED course were required to achieve a minimum of AAB at A level to include Biology and two other subjects and a minimum of five GCSEs at grade B to include English Language and Mathematics and two sciences. For 2012 entry, this increased to AAA at A level, with a fourth AS subject at grade B and a minimum of six GCSEs at grade A to include English Language and Maths and two sciences. All students were also required to take the UCAT, used as a part of the selection process during these years.

Please note the application requirements and processes for all the courses detailed above do not reflect the current admissions criteria.

### Data

The data used in this study use the following criteria:

All students starting between 2007 and 2012, based on the commencement date contained in The Higher Education Statistics Agency (HESA) student record [28]. It is not possible for students starting after 2013 to have graduate outcome data in UK Medical Education Database (UKMED), as the latest year with outcome data available at the time of writing this paper is 2018.

**Fig. 1:**
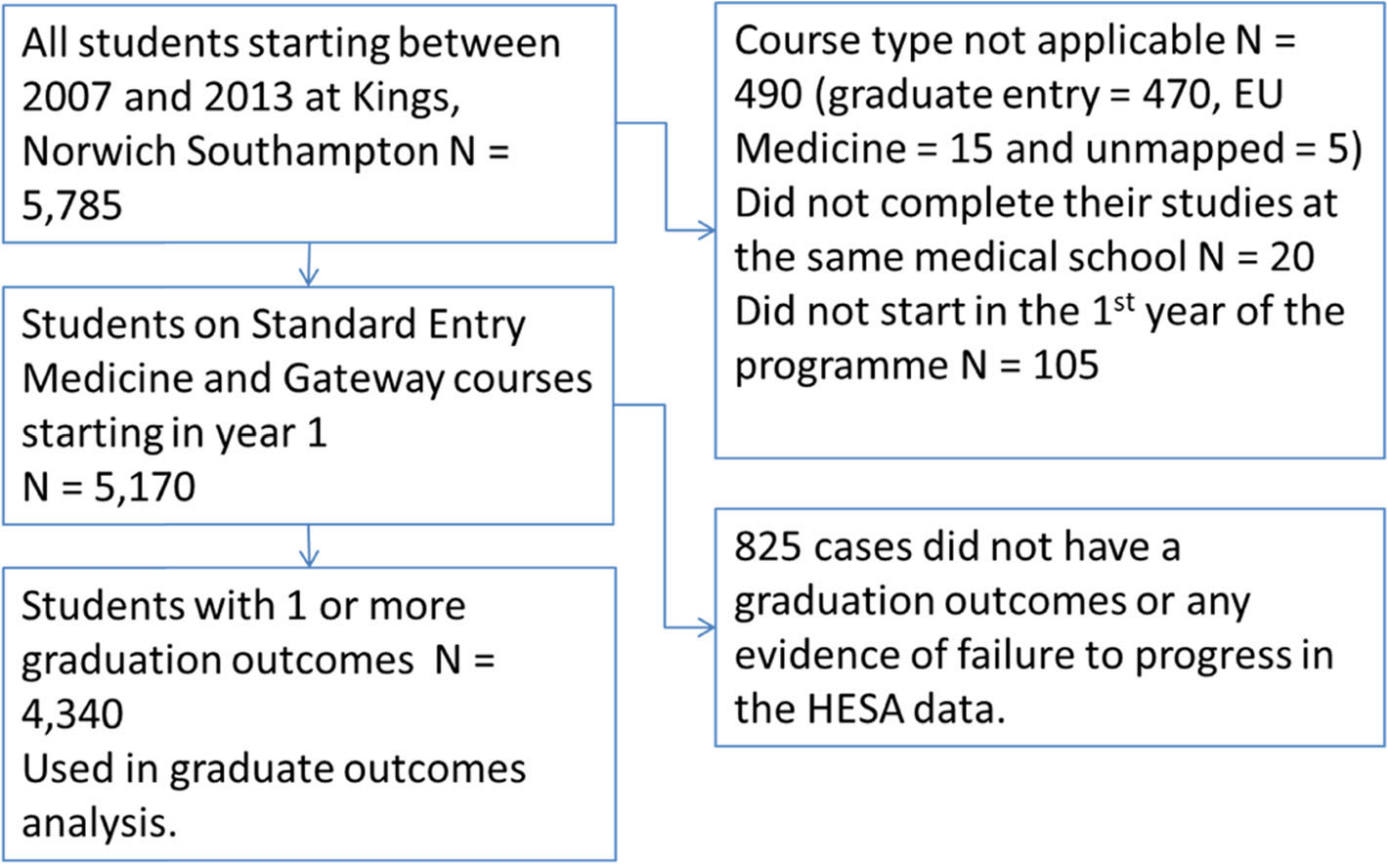
Flow of data through the study

Figure 1 shows the flow of data through the study, with the cases removed by exclusion criterion. Only cases where the first year of course was year 1 or year 0 were included in this analysis.

Five thousand one hundred seventy students completed their entire medical degree at the same medical school on either a SEMED or gateway course. 4340 of these students had one or more of the three outcomes on graduation and are included in the analysis.

### Measures

#### The EPM score

Students in a graduating cohort are ranked on their medical school performance (Educational Performance Measure, EPM). Individual schools decide which assessments to include in the EPM that meet the specified criteria, and are required to consult with students and publish on their website the assessments included in that score [29].

From 2013, all Foundation Programme applicants were ranked within their year and medical school into EPM deciles. In 2012, applicants were ranked into quartiles. To allow cases from 2012 and the later years to be combined in one analysis, EPM quartiles and deciles were converted to normal deviate scores – the approach taken by Garrud and McManus [30].

To include students who did not complete their studies, an 11th category was created consisting of all students who were recorded by HESA as leaving due to ‘Academic failure/left in bad standing/not permitted to progress’ in any academic year [31]. This approach gave 3840 cases with a normal deviate based on the decile, 275 based on the quartile and 200 in the 11th category of 0. We took the final attempt as the most valid due to the nature of the calculation.

#### The PSA score

The British Pharmacological Society developed the Prescribing Safety Assessment (PSA) jointly with the Medical Schools Council, with the aim of enabling students to demonstrate competencies in relation to the safe and effective use of medicines [32]. The PSA has been delivered from 2014. In 2014 and 2015, the PSA was a formative assessment at all three medical schools. From 2016, students have been required to pass before starting Foundation Year 2. The analysis uses the score relative to the pass mark converted to Z-scores. Z-scores were calculated from the mean and standard deviation for all attempts at the PSA for that year UK-wide (i.e. all cases in UKMED, not the extract used for this study).

#### The SJT score

The Situational Judgement Test (SJT) [33] is a final year undergraduate test that assesses individuals’ reactions to a number of hypothetical role-relevant scenarios, which reflect situations candidates are likely to encounter as a doctor. It seeks to provide a reliable measurement of the following non-academic domains: Coping with pressure, Working effectively as part of a team, Effective communication, Problem solving, and Commitment to professionalism.

The SJT raw score obtained from the Foundation Programme Application System was converted to a Z-score, based on the published mean and standard deviation from the technical report for the applicable year [34].

#### Attempts

The first score for both the PSA and the SJT, where there was more than one attempt, was used in these analyses. McManus and Ludka analysed Membership of the Royal Colleges of Physicians of the United Kingdom (MRCPUK) data and found that the mark at the first attempt of taking an examination is the best predictor of future performance and so is the most accurate measure of future performance [35].

#### A-levels

Points were calculated using the methods described in the UCAT-12 study: A = 10, B = 8, C = 6, D = 4, E = 2 [16]. To account for A* grades being introduced in 2010, the 3 best A-level score was expressed as a Z-score calculated within the year of qualification across all cases. In addition, a flag to indicate whether an A* grade was possible was derived from the qualification year, this was included in the regression models to control for the change in A-level scoring. There were only 21 cases with Scottish Qualifications Authority (SQA) qualifications and these were removed so only English qualifications are included in this study.

#### UCAT score

The UCAT is a cognitive skills test used by many UK medical schools in conjunction with other admissions processes to select students with the appropriate attributes to study medicine [36]. A UCAT total Z-score was calculated using the UCAT score associated with their entry to medical school (i.e. their final attempt if they had more than one), was calculated using the applicant test statistics published by UCAT for that year of entry [37].

## Results

The entry profiles of the 4340 cases with at least one graduation outcome were compared across the two course types for the three medical schools. Throughout the results HESA’s statistical disclosure controls have been applied as required by the UKMED research process [38]: All Ns are reported to the nearest 5. Ns with a denominator of less than 22.5 are not reported.

### Demographic data

Table 1 shows that students on gateway courses are significantly more likely (*p* <  0.001) than students on SEMED courses to be from an ethnic minority group; have studied at a state-funded school; have parents who have not gained higher education qualifications; live in an area with lower participation rates in higher education; be from a lower socio-economic group; have applied for a UCAT bursary, and live in a more deprived area.

**Table 1:** Summary of demographic data (*N* = 4340)

		Gateway	SEMED	Test of association	
Factor	Category	N (%)	N (%)	Χ^2^	P – Bonferroni correction applied
Sex	Male	250 (44.4)	1610 (42.5)	0.65	1
	Female	310 (55.6)	2175 (57.5)		
Ethnicity groups	BME	365 (65.2)	1595 (42.2)	105.1	< 0.001
	White	195 (34.6)	2160 (57.1)		
	Missing	0	30 (0.7)		
Disability	Not known	535 (95.9)	3500 (92.5)	8.08	0.04
	1 or more recorded by HESA	25 (4.1)	285 (7.5)		
School Type (HESA)	From state-funded school	515 (92.5)	2285 (60.4)	220.4	< 0.001
	Privately funded school	20	970 (25.6)		
	Missing	25 (4.1)	530 (14.0)		
Parental Education (at Higher Education)	No	315 (56.6)	690 (18.3)	422.5	< 0.001
	Yes	175 (31.7)	2685 (71.0)		
	Unknown	65 (11.6)	405 (10.8)		
Participation of local areas (POLAR)	1	55 (9.50)	135 (3.6)	268.0	< 0.001
	2	100 (18.10)	260 (6.9)		
	3	150 (26.7)	485 (12.8)		
	4	120 (21.9)	855 (22.6)		
	5	115 (20.4)	1555 (41.1)		
	Missing	20	490 (13.0)		
Index of multiple deprivation (IMD)	1 - Most deprived	210 (37.8)	225 (5.9)	672.0	< 0.001
	2	110 (19.9)	395 (10.4)		
	3	95 (17.20	620 (16.4)		
	4	50 (9.3)	825 (21.8)		
	5 - Least deprived	70 (12.4)	1230 (32.5)		
	Missing	20	490 (12.9)		
Socioeconomic classification (SEC)	Semi-routine and routine occupations	140 (24.9)	295 (7.8)	277.7	< 0.001
	Lower supervisory and technical occupations	20	65 (1.8)		
	Small employers and own account workers	40 (7.5)	200 (5.3)		
	Intermediate occupations	55 (9.9)	345 (9.1)		
	Managerial and professional occupations	235 (41.8)	2710 (71.7)		
	Unknown	70 (12.5)	165 (4.3)		
UCAT Bursary	No	430 (76.9)	3625 (95.8)	279.5	< 0.001
	Yes	130 (23.1)	160 (4.2)		

### Educational attainment on entry to medical school

Table 2 summarises educational prior attainment and test scores on entry. In accordance with the entry criteria, students on the gateway courses had lower A-level points and lower UCAT test scores than those on SEMED courses. There were large differences on entry between the two groups: Cohen’s *d* = 1.254 (Z-score A-level best 3) and Cohen’s *d* = 1.066 (Z-score UCAT total). Using the 4340 cases with a graduate outcome as a starting point UCAT data were missing in 125 cases and A-level data in 880 cases.

**Table 2:** Educational Attainment and UCAT scores on entry to medical school

Variable	Derivation	Gateway			SEMED			Total				
		Mean	Std. Dev	N	Mean	Std. Dev	N	Mean	Std. Dev	N	F	P – Bonferroni
A level Total Points	Total Score of all A-Levels taken, excluding General Studies.	24.32	4.319	490	29.63	4.618	2975	28.877	4.9376	3465	568.36	0.00
Z score in year of qualification for the 3 best A-levels	Z-score of the total score of the 3 best A levels within a HESA qualification year	−0.898	0.885	490	0.231	0.7970	2970	0.0708	0.901	3460	821.5	0.00
UCAT TOTAL score		2384.8	230.25	540	2633.9	217.2	3675	2601.9	234.2	4215	611.0	0.00
UCAT TOTAL Z-score	Z-score using mean and SD from the admission year for all candidates	− 0.212	0.837	540	0.6916	0.785	3665	0.5756	0.848	4205	612.3	0.00

### Comparison of outcomes measures on graduation

#### EPM deviate score

**Fig. 2:**
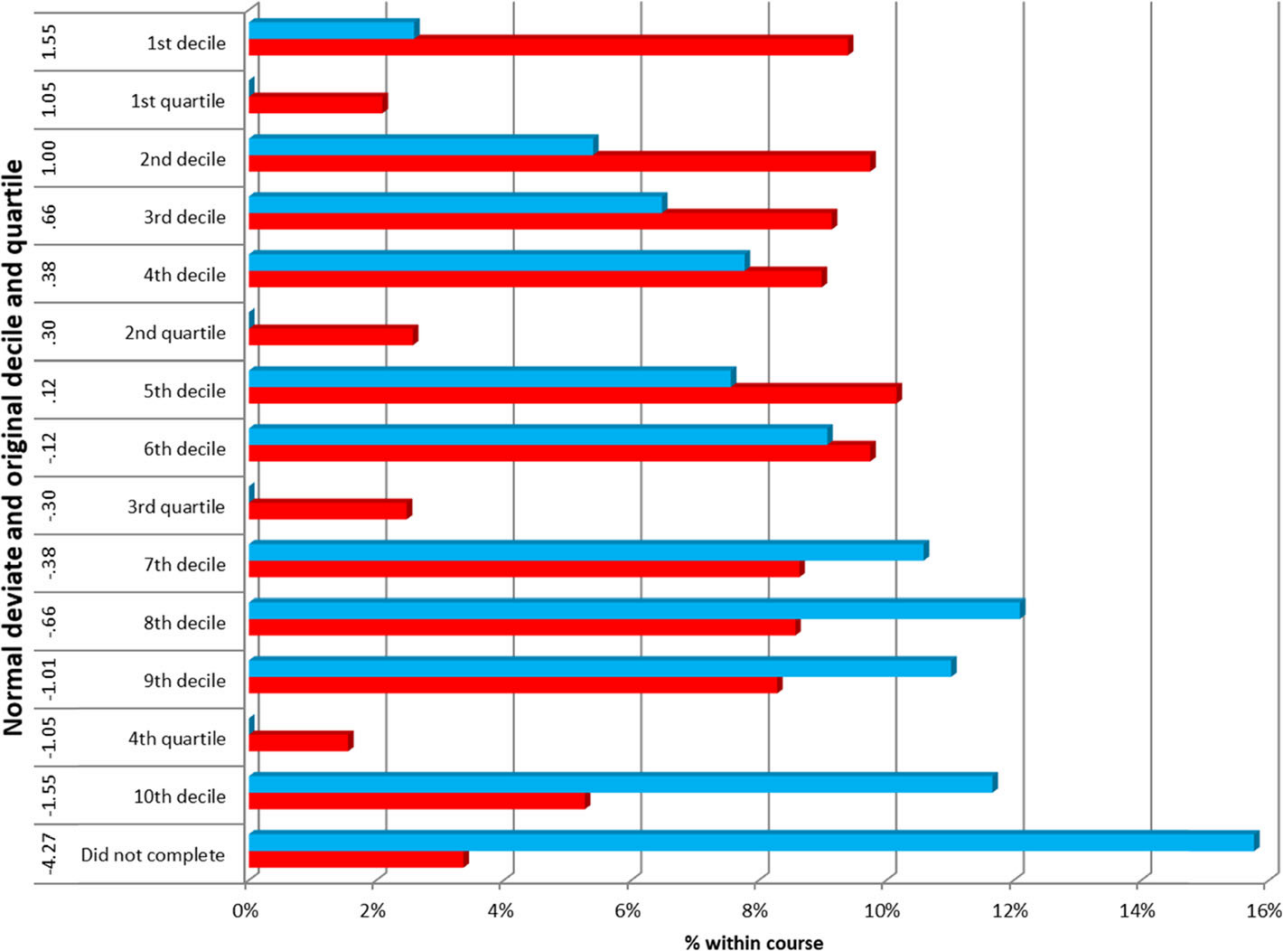
Distribution of EPM Normal Deviate Score by Course Type (*N* = 4310)

Figure 2 shows the proportion of students within each EPM Normal deviate by course type. There were 3760 cases from SEMED and 555 from gateway courses. (Missing data in 30 cases, all cases with a PSA score and therefore included, but no EPM or SJT data. All 30 had gained their PMQ.) The mean EPM Normal Deviate score for SEMED students was −.0350 (SD = 1.121, *N* = 3760); for gateway students it was − 0.883 (SD = 1.591, *N* = 555) Cohen’s *d* = 0.692.

#### Predicting EPM Normal deviate score

In Table 3 the results are presented for two multiple regressions predicting EPM scores. In the 1st model only school and course type are included with Norwich set arbitrarily as the reference category for medical school, and gateway for course type; in the 2nd model A-levels and UCAT scores on entry are included to control for attainment on entry to the course.

**Table 3:** Predicting EPM from course type, academic attainment and aptitude scores on entry to medical school

		Unstandardized Coefficients		Stand. Coeff.	t	Sig.	95% Confidence Interval for B		*sr*^*2*^
		B	Std. Error	Beta			Lower Bound	Upper Bound	
EPM Deviate – model 1	(Constant)	−0.812	0.066		−12.214	0.000	−0.942	− 0.681	
	King’s	−0.227	0.053	−0.092	−4.281	0.000	−0.331	−0.123	0.005
	Southampton	−0.037	0.057	−0.014	−0.656	0.512	−0.148	0.074	0.000
	SEMED	0.893	0.059	0.254	15.236	0.000	0.778	1.008	0.064
EPM Deviate – model 2	(Constant)	−0.528	0.075		−7.031	0.000	−0.675	− 0.381	0.000
	King’s	−0.377	0.055	−0.153	−6.832	0.000	−0.485	− 0.268	0.013
	Southampton	−0.086	0.056	−0.033	−1.522	0.128	−0.196	0.025	0.001
	SEMED	0.542	0.070	0.154	7.769	0.000	0.405	0.679	0.016
	Z UKCAT total	0.171	0.029	0.114	5.867	0.000	0.114	0.228	0.009
	Z in year of qual. A-level best 3	0.163	0.025	0.120	6.410	0.000	0.113	0.212	0.011
	A* A-level possible	−0.012	0.041	−0.005	−0.292	0.770	− 0.093	0.069	0.000

EPM deviate model 1 (*R*^2^ adj = 0.072. *N* = 3350, *P* <  0.001) shows the relationship between the course type and EPM deviate score. SEMED students are significantly more likely to have a higher score with course type accounting for 6.4% of the variance in the EPM normal deviate score.

EPM deviate model 2 (*R*^2^ adj = 0.094. N = 3350, *P* < 0.001) shows that course type is still significant but only accounts for 1.6% of the variance once the measures of attainment and aptitude on entry are included. Therefore, there is less difference between EPM by course type when taking prior attainment and aptitude into account.

**Fig. 3:**
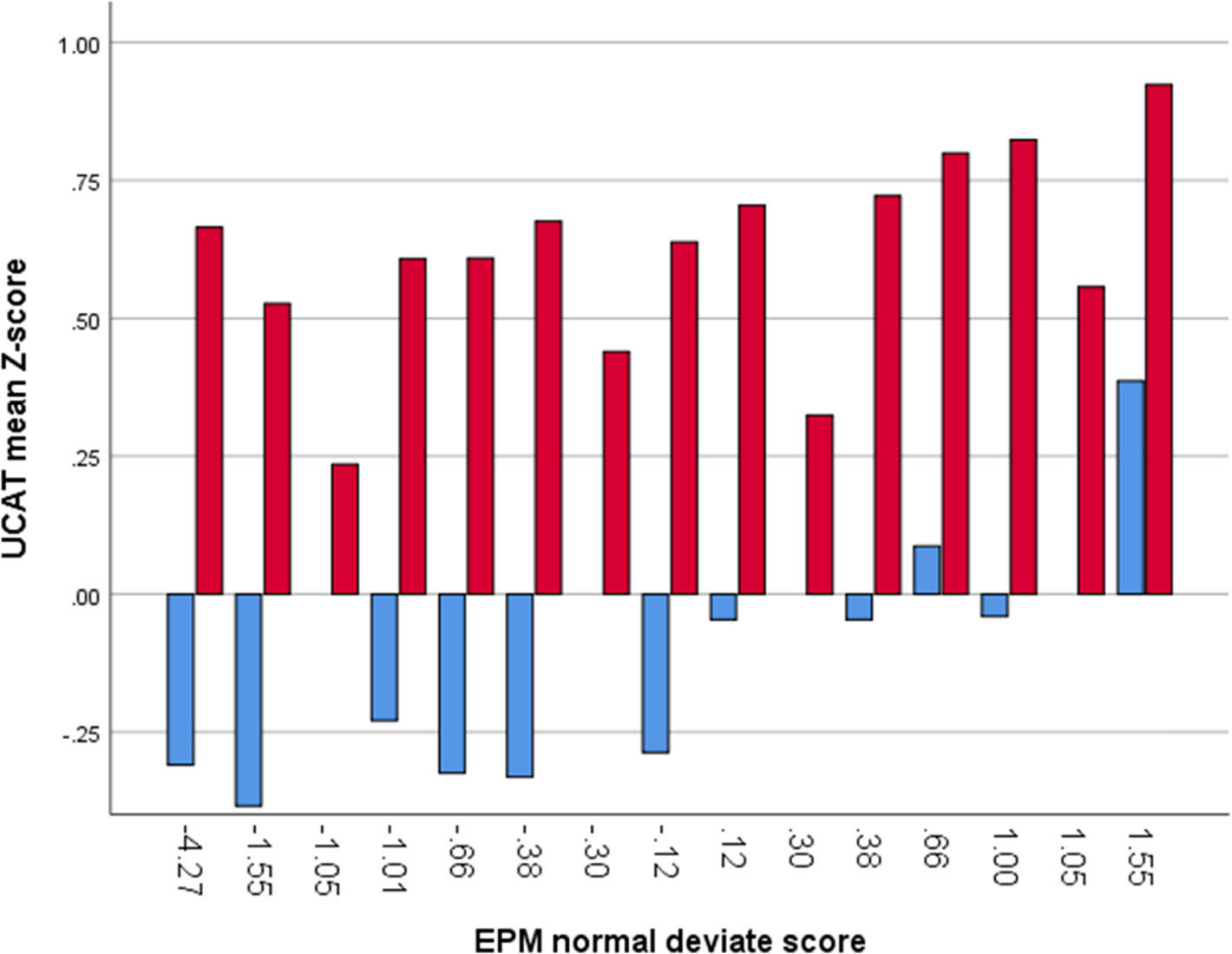
UCAT total as Z-scores by EPM Normal Deviate score

**Fig. 4:**
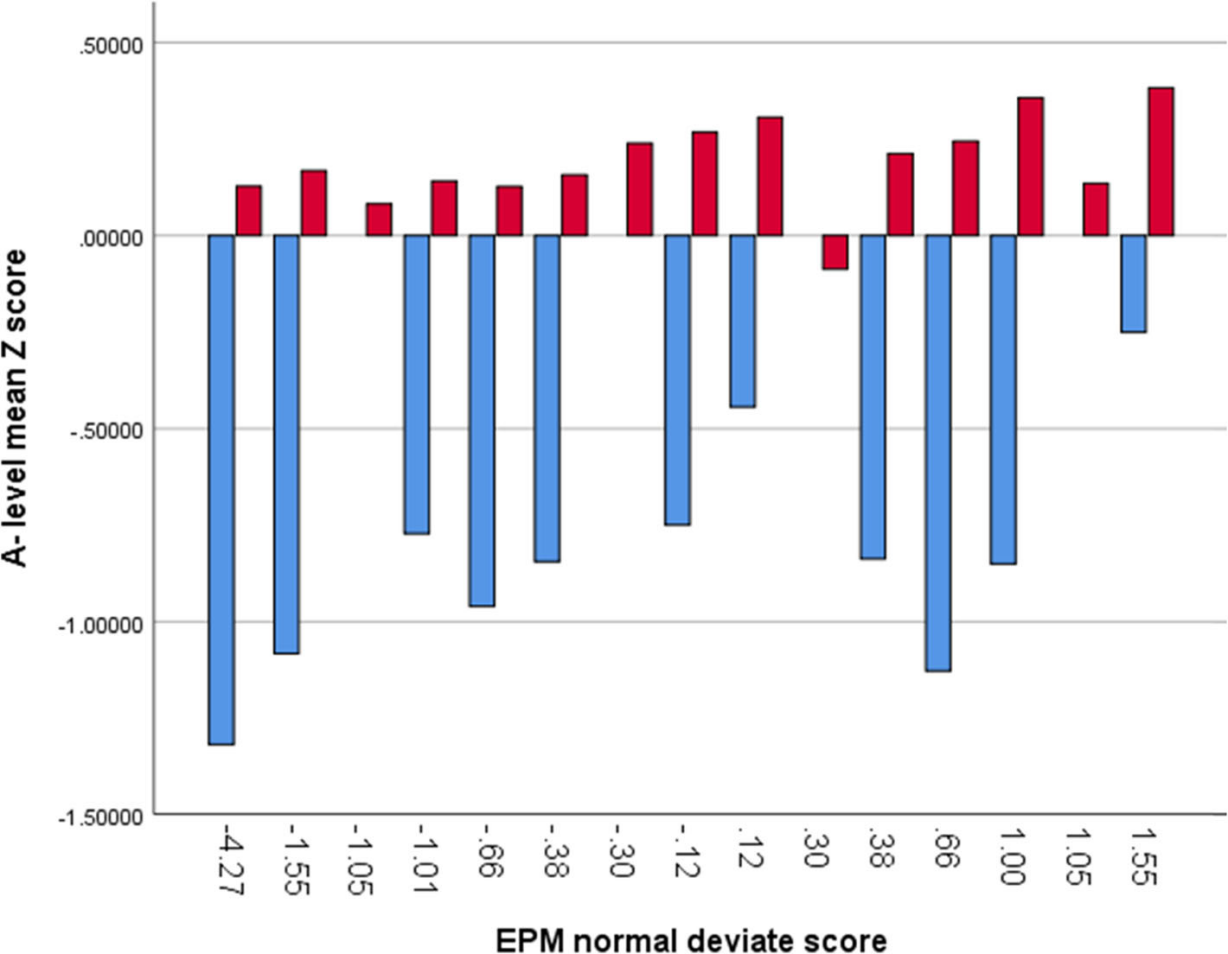
Academic attainment as Z-scores by EPM Normal Deviate score

Figures 3 and 4 - Gateway students show a wide distribution across EPM normal deviate scores, especially in relation to A levels, despite having significantly lower attainment and aptitude on entry. It is notable that gateway students in the top decile also had the highest UCAT scores

#### Predicting PSA and SJT scores

As seen in Table 4, SEMED students have significantly higher mean PSA z-scores. The majority of cases with no PSA score graduated before the PSA exam was introduced. The earliest PSA exam was held in February 2014 so there are no PSA scores for the 862 cases that graduated in 2012 and 2013.

**Table 4:** PSA z-scores by course type

	SEMED			Gateway			Total			F	P
	Mean	Std. Dev	N	Mean	Std. Dev	N	Mean	Std. Dev	N		
PSA	−0.117	0.958	2805	−0.746	1.084	410	−0.198	0.997	3215	149.48	< 0.001

In Table 5 results are presented for two multiple regressions predicting PSA. In the 1st model only school and course type are included with Norwich set arbitrarily as the reference category for medical school, and gateway for course type; in the 2nd model A-levels and UCAT scores on entry are included to control for attainment on entry to the course.

**Table 5:** Predicting PSA z-scores from scores on entry to medical school and course type

		Unstandardized Coefficients		Stand. Coeff.	t	Sig.	95% Confidence Interval for B		*sr*^*2*^
		B	Std. Error	Beta			Lower Bound	Upper Bound	
PSA									
PSA Model 1	(Constant)	−0.446	0.061		−7.282	0.000	−0.566	−0.326	0.000
	King’s	−0.291	0.049	−0.145	−5.956	0.000	−0.387	−0.195	0.012
	Southampton	−0.613	0.053	−0.283	−11.642	0.000	−0.716	−0.509	0.047
	SEMED	0.658	0.053	0.230	12.323	0.000	0.553	0.763	0.053
PSA Model 2	(Constant)	−0.264	0.078		−3.371	0.001	−0.417	− 0.110	0.000
	King’s	−0.460	0.050	−0.229	−9.170	0.000	−0.559	−0.362	0.028
	Southampton	−0.661	0.052	−0.306	−12.827	0.000	−0.763	−0.560	0.054
	SEMED	0.295	0.078	0.103	3.783	0.000	0.142	0.448	0.005
	Z UKCAT total	0.250	0.027	0.200	9.233	0.000	0.197	0.303	0.028
	Z in year of qual. A-level best 3	0.121	0.023	0.109	5.327	0.000	0.077	0.166	0.009
	A* A-level possible	0.196	0.098	0.097	1.996	0.046	0.003	0.388	0.001
	Interaction: course * Z in year of qual. A-level best 3	−0.085	0.106	−0.042	−0.803	0.422	−0.292	0.122	0.000

The 2nd model shows students on a SEMED course are more likely to achieve a high PSA z-score even after controlling for aptitude and attainment on entry to the course. PSA Model 1 (*R*^*2*^ *adj* = 0.102. *N* = 2580, *P* < 0.001) shows that the association between course type and PSA result is greater without including the measures of prior attainment and aptitude on entry, but their inclusion in PSA Model 2 (*R*^*2*^ *adj =* 0.149. N = 2580, *P* < 0.001) does not account for all the variance associated with course type in the first model. In the 1st Model course type account 5.3% (*sr*^*2*^) of the variance; in the 2nd it accounts for less than 1%.

Table 6 shows there is a smaller difference in SJT z-scores between SEMED and gateway students than seen for the PSA.

**Table 6:** SJT z-scores by course type

	SEMED			Gateway			Total			F	P
	Mean	Std. Dev	N	Mean	Std. Dev	N	Mean	Std. Dev	N		
SJT	0.891	0.312	3785	0.844	0.363	560	0.885	0.320	4340	10.31	0.001

In Table 7 the SJT Model 1 (*R*^*2*^ *adj =* 0.008. *N* = 3375, *P* < 0.001) shows there is a difference in SJT z-scores by course type, with those on standard entry courses scoring more highly. However, this is no longer the case when the UCAT and A-level points are included in SJT Model 2 (*R*^*2*^ *adj =* 0.035. N = 3375, P < 0.001) showing that when controlling for attainment of entry there is no difference across the two course types or the three schools on SJT z-scores.

**Table 7:** Predicting SJT z-scores from scores on entry to medical school and course type

			95% Confidence Interval for B		Sig.	*sr*^*2*^
		B	Lower Bound	Upper Bound		
SJT Model 1	(Constant)	0.825	0.792	0.858	0.000	0.000
	King’s	0.032	0.006	0.059	0.016	0.002
	Southampton	0.002	−0.027	0.030	0.913	0.000
	SEMED	0.067	0.038	0.096	0.000	0.006
SJT Model 2	(Constant)	0.887	0.845	0.930	0.000	0.000
	King’s	0.016	−0.012	0.043	0.259	0.000
	Southampton	−0.008	−0.036	0.020	0.579	0.000
	SEMED	−0.039	−0.081	0.003	0.070	0.001
	Z UCAT total	0.006	−0.008	0.021	0.411	0.000
	Z in year of qual. A-level best 3	0.025	0.012	0.038	0.000	0.004

**Fig. 5:**
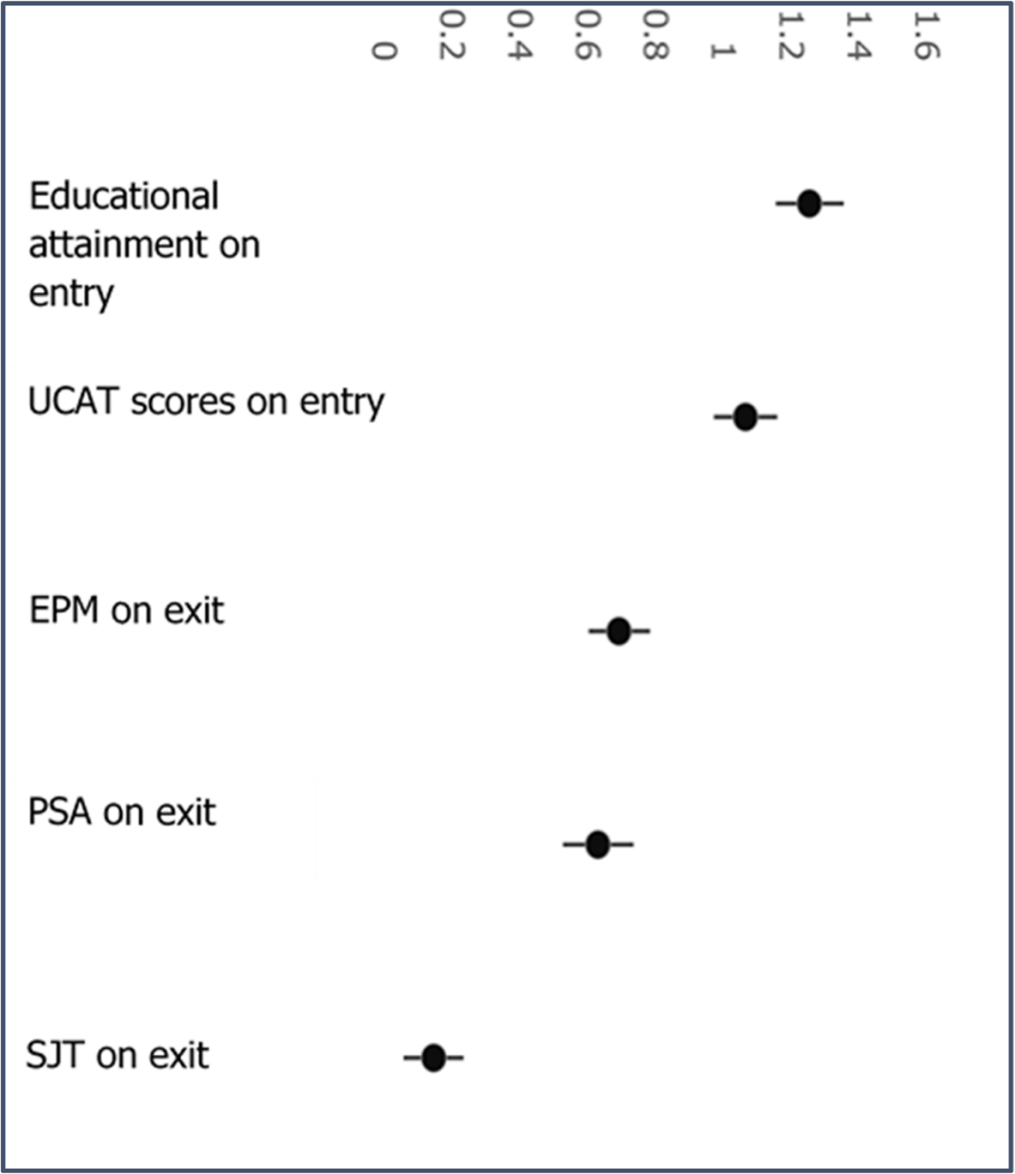
Differences between SEMED and gateway on the entry and exit measures with 95% confidence intervals

Figure 5 shows the Cohen’s *d* for the measures on entry and exit: There is a smaller difference on exit (EPM Cohen’s *d* = 0.616; PSA Cohen’s d = 0.631; SJT Cohen’s *d* = 0.1454) than seen on entry (A level Cohen’s d = 1.254 and UCAT Cohen’s *d* = 1.066) between the two groups. The confidence intervals are calculated using a method described by Hedges and Olkin [39].

#### Attrition during medical school

Table 8 - The 1st academic year of each case’s student record has been used as the starting point and called 1st AC year, regardless of whether this was coded to Year of Programme = 0 or Year of Programme = 1 in the HESA data. Apart from Southampton, gateway courses are not consistent over time in their use of Year of Programme = 0 coding for the first academic year of study on a gateway programme, so this approach allows a consistent methodology. Attrition is higher in the 2nd and 3rd academic years of the gateway course; for SEMED attrition is highest in the 2nd academic year.

**Table 8:** Attrition of students on gateway and standard entry courses – (cases included in EPM analysis *N* = 4310 Fig. 2)

	No academic failure recorded and no PMQ obtained	Academic failure/left in bad standing/ not permitted to progress - 1st AC YEAR	Academic failure/left in bad standing /not permitted to progress - 2nd AC YEAR	Academic failure/left in bad standing/ not permitted to progress - 3rd AC YEAR	Academic failure/left in bad standing/ not permitted to progress - 4th AC YEAR	Academic failure/left in bad standing/ not permitted to progress - 5th AC YEAR	Academic failure/left in bad standing/ not permitted to progress - 6th AC YEAR	Academic failure/left in bad standing/ not permitted to progress - 7th AC YEAR	Has PMQ and no failure to progress recorded	Total
Gateway	10	5	30	20	15	5	0	0	460	555
	2.20%	1.10%	5.40%	4.00%	2.90%	1.10%	0.40%	0.00%	83.00%	100.00%
Standard Entry Medicine	30	15	65	25	10	0	0	0	3615	3760
	0.70%	0.50%	1.80%	0.60%	0.20%	0.00%	0.00%	0.10%	96.10%	100.00%
Total	40	25	95	45	25	5	5	0	4070	4310
	0.90%	0.50%	2.20%	1.00%	0.60%	0.20%	0.10%	0.00%	94.40%	100.00%

Additional analysis showed gateway students were more likely to repeat a year (Χ^2^ = 3226.5, *P* < 0.001) with 75.2% (*N* = 2420) of SEMED students, who did not take an intercalated BSc degree, completing in 5 years compared to 62.1% (*N* = 295) of gateway students, who did not take an intercalated BSc or MSc degree, completing in 6 years.

## Discussion

This is the first study to provide a multi-institution picture of progression and performance of students studying on long-standing gateway courses in the UK. Although the proportion of students entering medical school through these routes is small, it is growing, with the number of gateway courses increasing from three in 2007 to nineteen in 2020. The results of this study confirm such courses are successful in increasing undergraduate diversity, with gateway students being significantly more likely to have demographic characteristics associated with low socioeconomic status than SEMED students.

As the intention of gateway courses is to widen participation in medicine these results may not appear surprising, however, the majority of the demographic variables presented in this paper are not eligibility criteria required for entry on to these courses. Therefore, this analysis provides some cross-validation that gateway courses are successfully attracting students from under-represented, low socio-economic backgrounds, through a variety of contextual measures. Students on gateway courses are also significantly more likely to have lower A-level grades and UCAT test scores than those on SEMED courses. These are expected findings and in accordance with the admissions and academic entry criteria for these courses.

When comparing graduation outcomes, SEMED students score more highly in all assessments. However, these differences reduce or, in the case of SJT, disappear after controlling for attainment on entry. A higher UCAT total score, higher A-level points and undergoing a standard entry medicine course were all associated with a higher EPM and PSA scores, which supports the findings of McManus et al., although their reported outcomes did not specifically look at EPM, PSA or SJT [24]. The results of this study also align with MacKenzie et al. who reported the UCAT total score is predictive of performance on the EPM and the SJT [25]. The difference seen in SJT and EPM outcomes after adjusting for attainment and aptitude may reflect that the SJT has less variation at the top end of ability. As Smith and Tiffin note: “SJT scores are likely to be relatively poor at differentiating more highly performing candidates from each other.” [40]

The wide distribution of gateway EPM normal deviate scores indicates that despite significantly lower attainment and aptitude on entry, gateway students are achieving scores across all deciles. This supports the recommendation by Mwandigha et al. of a reduction in academic entry criteria for students from lower performing secondary schools [15].

The authors acknowledge the data used on entry and exit are not directly comparable, however, both include established and robust components designed to assess academic attainment and aptitude for study in the medical profession. In this context, the smaller difference in performance seen for the outcomes compared to initial attainment and aptitude on entry between the cohorts presents modest evidence that gateway courses provide students with an opportunity to demonstrate greater academic potential than their secondary educational attainment would suggest. Despite gateway courses providing such opportunities, it is unrealistic to expect they will effectively remove all disadvantage and ‘level the playing field’, so it is important to consider these findings in the wider context of WP.

Gateway courses offer contextual admissions to students who have experienced social and educational disadvantage. For many of these students, the context of their disadvantage does not disappear when they enter medical school. Many factors that have prevented them from reaching their academic potential will continue to affect their ability to study optimally and, for a few, to progress through medical school to graduation. A recent study showed medical students from a WP background had less choice regarding when and how often they undertook paid employment, which negatively affected their studies and exam preparation. In addition, half of the WP students interviewed worked to support their families as well as themselves [41]. Such additional, and on occasion, unsustainable pressures may help explain the greater rate of attrition for students on gateway courses, also reported in previous single site studies [20, 21].

Success is frequently associated with high academic achievement in medicine, but for any student who has competing interests on their time, such as paid employment or family and caring responsibilities, graduating from medical school irrespective of scores and rankings is a success. Postgraduate success of doctors who studied on gateway courses has yet to be established as too few have completed their postgraduate training and it remains to be seen how the proportion of gateway graduates joining the GP and Specialist registers compares to those from SEMED courses. Future research will follow the cohorts reported here until the completion of their postgraduate training to establish their career progression.

## Conclusions

Overall, this study provides evidence that gateway courses are proving successful in the undergraduate arena, with many students thriving academically and with the majority graduating as doctors. It is also clear that gateway courses are achieving their recruitment aims with students on gateway courses being significantly more likely to have demographic characteristics associated with low socioeconomic status. Clear evidence of differential attainment in gateway students on entry and exit from medical school is reported. However, the difference between gateway and SEMED students seen in the outcome measures compared to attainment and aptitude on entry is smaller, providing modest evidence that gateway courses are helping students realise their academic potential. It is important that the attainment gap and greater attrition rate reported in this paper are contextualized within the expectations and realities of being a widening participation student. These findings show further research is necessary to inform the development of appropriate support for students on gateway courses. The new and emerging culture of WP and gateway courses will require time to embed within medical schools, ensuring the curriculum and support processes are fully inclusive and appropriate to the needs of all students, optimising their success. It will take quite a few generations until there are a proportionate number of doctors from under-represented backgrounds who can act as relatable role models and effectively diversify the workforce, creating an more inclusive environment.

## Data Availability

Source of data was the UK Medical Education Database (“UKMED”) UKMEDP38 extract generated on 27th February 2019 and Approved for publication on 20th December 2018. The final extract was produced after the report had been approved as we re-ran the analyses with an additional year’s worth of data. We are grateful to UKMED for the use of these data. However, UKMED bears no responsibility for their analysis or interpretation. The data includes information derived from that collected by the Higher Education Statistics Agency Limited (“HESA”) and provided to the GMC (“HESA Data”). Source: HESA Student Record [2002/2003 and 2016/2017] Copyright Higher Education Statistics Agency Limited. The Higher Education Statistics Agency Limited makes no warranty as to the accuracy of the HESA Data, cannot accept responsibility for any inferences or conclusions derived by third parties from data or other information supplied by it. Applications to view the data can be made to UKMED.

## Abbreviations

BMA: British medical association
EMA: Educational maintenance allowance
EMDP: Extended medical degree programme
EPM: Educational performance measure
GMC: The general medical council
HE: Higher education
HESA: Higher education statistics agency
IMD: Index of multiple deprivation
MMIs: Multiple-mini interviews
MRCPUK: Membership of the royal colleges of physicians of the United Kingdom
MSC: Medical schools council
NHS: National health service
PSA: Prescribing safety assessment
SEMED: Standard entry medical degree
SES: Socioeconomic status
SJT: Situational judgement test
SQA: Scottish qualifications authority
UCAT: Universities clinical aptitude test
UKMED: UK medical education database
WP: Widening participation
